# Herbivory drives large-scale spatial variation in reef fish trophic interactions

**DOI:** 10.1002/ece3.1310

**Published:** 2014-11-22

**Authors:** Guilherme O Longo, Carlos Eduardo L Ferreira, Sergio R Floeter

**Affiliations:** 1 Programa de Pós-Graduação em Ecologia, Universidade Federal de Santa CatarinaFlorianópolis, Brazil; 2 Laboratório de Biogeografia e Macroecologia Marinha, Departamento de Ecologia e Zoologia, Universidade Federal de Santa CatarinaFlorianópolis, Brazil; 3 Laboratório de Ecologia e Conservação de Ambientes Recifais, Universidade Federal FluminenseNiterói, Brazil

**Keywords:** Brazil, critical functions, feeding pressure, functional groups, geographic variation

## Abstract

Trophic interactions play a critical role in the structure and function of ecosystems. Given the widespread loss of biodiversity due to anthropogenic activities, understanding how trophic interactions respond to natural gradients (e.g., abiotic conditions, species richness) through large-scale comparisons can provide a broader understanding of their importance in changing ecosystems and support informed conservation actions. We explored large-scale variation in reef fish trophic interactions, encompassing tropical and subtropical reefs with different abiotic conditions and trophic structure of reef fish community. Reef fish feeding pressure on the benthos was determined combining bite rates on the substrate and the individual biomass per unit of time and area, using video recordings in three sites between latitudes 17°S and 27°S on the Brazilian Coast. Total feeding pressure decreased 10-fold and the composition of functional groups and species shifted from the northern to the southernmost sites. Both patterns were driven by the decline in the feeding pressure of roving herbivores, particularly scrapers, while the feeding pressure of invertebrate feeders and omnivores remained similar. The differential contribution to the feeding pressure across trophic categories, with roving herbivores being more important in the northernmost and southeastern reefs, determined changes in the intensity and composition of fish feeding pressure on the benthos among sites. It also determined the distribution of trophic interactions across different trophic categories, altering the evenness of interactions. Feeding pressure was more evenly distributed at the southernmost than in the southeastern and northernmost sites, where it was dominated by few herbivores. Species and functional groups that performed higher feeding pressure than predicted by their biomass were identified as critical for their potential to remove benthic biomass. Fishing pressure unlikely drove the large-scale pattern; however, it affected the contribution of some groups on a local scale (e.g., large-bodied parrotfish) highlighting the need to incorporate critical functions into conservation strategies.

## Introduction

Trophic interactions are fundamental to the structure and function of ecosystems by altering patterns of species density and biomass across different trophic levels (Paine 1992). Anthropogenic activities are negatively affecting trophic interactions, causing severe changes in ecosystems, from biodiversity loss to shifts in abiotic conditions (Estes et al. 2011). Understanding the strength and distribution of trophic interactions in natural communities and their response to these changes is critical to support informed conservation actions (Duffy 2002).

Comparisons of trophic interactions along geographic scales can provide a broader understanding of their importance in changing ecosystems by benefitting from natural gradients, for example, when there is variation in the species richness or abiotic conditions (Pennings & Silliman 2005). A recent large-scale study spanning a 32° latitudinal gradient in sea grass beds demonstrated that predation on marine sessile invertebrate communities and resulting effects on species richness were stronger in the tropics compared to temperate regions (Freestone et al. 2011). However, in both terrestrial and marine systems, most large-scale comparisons of trophic interactions are focused on herbivory, without considering other trophic categories, and present inconsistent results (Moles et al. 2011; Poore et al. 2012).

In marine ecosystems, herbivory is widely recognized as a critical process (Poore et al. 2012), affecting the structure and functioning of different systems (e.g., rocky reefs – Sala & Bouderesque 1997; coral reefs – Mumby 2006; kelp forests – Carter, VanBlaricom & Allen 2007). Although the large-scale geographic variation of plant chemical defenses and susceptibility to herbivory have been investigated (e.g., Bolser & Hay 1996; Pennings, Siska & Bertness 2001), large-scale comparisons of the intensity of herbivory in marine systems through standardized methods are relatively uncommon (see Pennings & Silliman 2005; Pennings et al. 2009; Bennet & Bellwood 2011). A recent meta-analysis found little to no influence of temperature on herbivory in marine systems, but highlighted the strong effects herbivores have on producer's abundance (Poore et al. 2012).

A commonly referred hypothesis states that the ability of marine ectothermic herbivores to digest and assimilate plant material would decrease with lower temperatures (Gaines & Lubchenco 1982). This has been proposed as an explanation for the decrease in the species richness, abundance, and bite rates of tropical herbivorous reef fishes as latitude increases in the Atlantic (i.e., toward colder areas; Floeter et al. 2005). Conversely, a recent large-scale study comprising three sites spanning 11° of latitude on the coast of New Zealand argued that temperature is unlikely a constraint to temperate herbivorous fish because there were no differences in demographic patterns between herbivorous and carnivorous fishes from warmer and colder areas (Trip et al. 2013). Hence, there is still a debate on the interactive mechanisms between herbivory by reef fishes and temperature, with few studies going beyond patterns of species richness and abundance (see Bennett and Bellwood 2011).

Directly quantifying herbivory and predation as trophic interactions (e.g., rates of interaction) instead of inferring these rates through species richness and abundance across large spatial scales is challenging (Pennings & Silliman 2005; Freestone et al. 2011). Reef fishes feeding on the benthos comprise a good model to address such question, as these trophic interactions play an essential role in structuring benthic communities, for example, through herbivory and predation on mesograzer crustaceans (Lewis 1986; Duffy & Hay 2000; Ceccarelli, Jones & McCook 2001; Kramer, Bellwood & Bellwood 2013). To date, studies have mainly focused on macroalgal removal, relative richness, and abundance of herbivores or trophic structure of communities, often neglecting other trophic interactions rather than herbivory (Ferreira et al. 2004; Floeter et al. 2005; Bennett and Bellwood 2011; Cheal et al. 2013). However, the per capita effects among species rather than differences in species richness and abundances may be driving shifts in interaction strength and thus need to be assessed (Pennings & Silliman 2005).

Food webs are generally structured by a few disproportionately strong and several weak to intermediate interactions (Paine 1992). The dominance of a few species in a given ecological process results in low ecological redundancy, commonly associated with less stability to disturbances (Duffy 2002; Hoey & Bellwood 2009). As a result, higher species diversity may not equate to higher system stability when species perform functions unevenly (Duffy 2002; Hooper et al. 2005). Consumers that impact the ecosystem disproportionately to their abundances can play central roles in the structure and function of communities (Power et al. 1996). A reduction in functionally dominant trophic links can prompt declines in biodiversity, therefore identifying these central species across geographic scales could guide the conservation of key ecological processes they mediate (Paine 1992; Duffy 2002; Green & Bellwood 2009).

**Figure 1:**
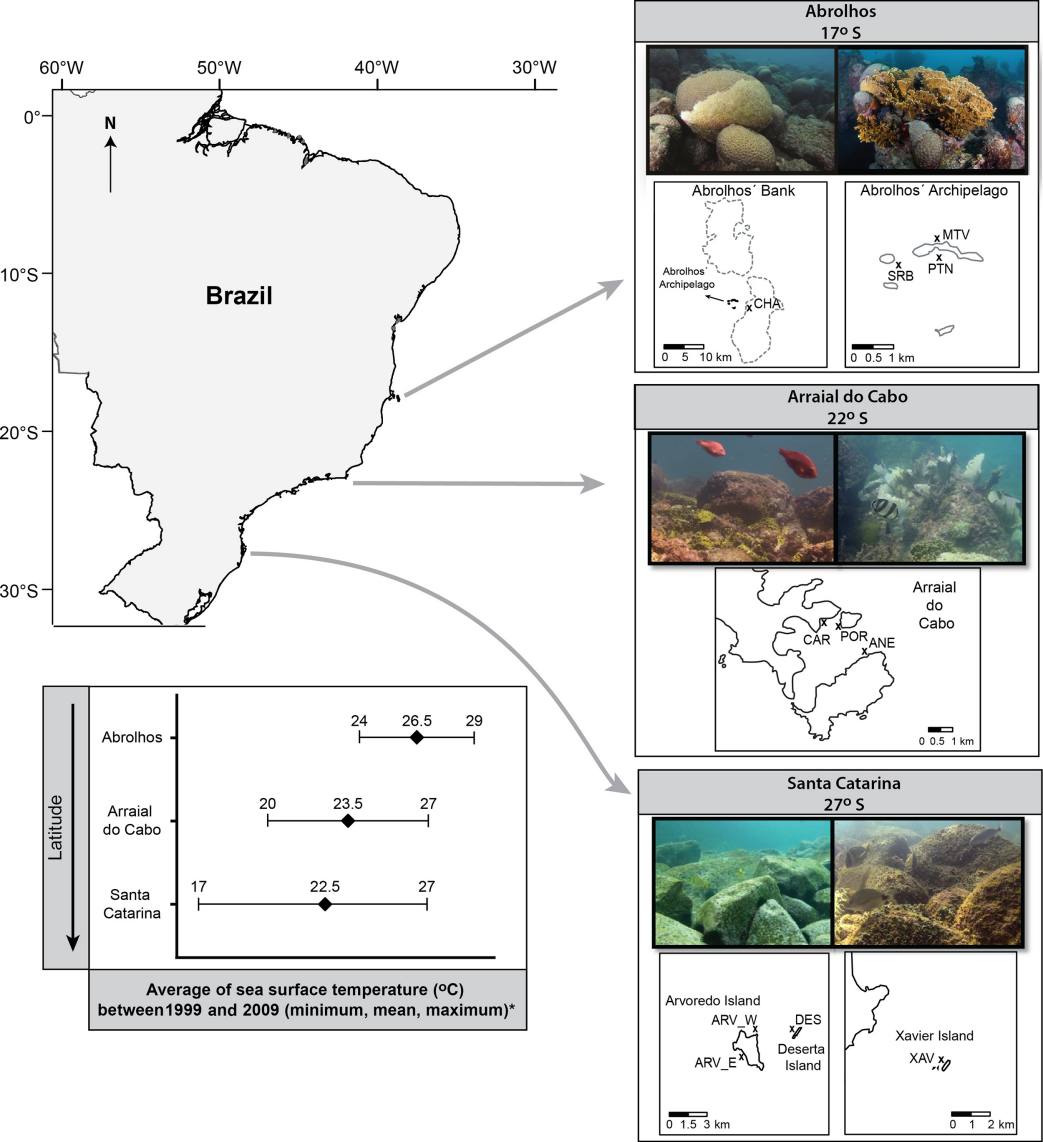
Studied reef areas along the Brazilian coast. Abbreviations in the maps indicate the locations within sites: CHAP, Chapeirão; MTV, Mato Verde; SRBA, Siriba; PTN, Portinho Norte; CAR, Cardeiros; POR, Porcos; ANE, Anequim; ARV_W, western Arvoredo; Arv_E, eastern Arvoredo; DES, Deserta; XAV = Xavier. * Sea surface temperature data from NOAA (http://www.nodc.noaa.gov/sog/cortad). Photographs: J.P. Krajewski and G.O. Longo.

We explored the large-scale variation of reef fish feeding pressure on the benthos in three sites spanning 10° of latitude, encompassing tropical and subtropical reefs along the Brazilian coast (Fig.1). Feeding pressure was determined combining bite rates on the substrate and the individual biomass per unit of time and area. This study aims to: (i) determine how total feeding pressure and the contribution of different fish functional groups within distinct trophic categories vary across large spatial scales; and (ii) identify species and functional groups that perform higher feeding pressure than predicted by their abundances, highlighting their importance to the ecosystem and the need for incorporating functional approaches into conservation strategies.

## Materials and Methods

### Study area

This study was conducted in three reef sites along the Brazilian coast, encompassing tropical and subtropical reefs from 17°S to 27°S, with mean annual sea surface temperature varying from 26.5°C to 22.5°C, respectively (Fig.1; average annual temperatures from 1999 to 2009 available in http://www.nodc.noaa.gov/sog/cortad/; NOAA). In each site, a minimum of three locations were sampled during the austral summer to account for local heterogeneity and all fieldwork was staggered between 09:00 and 15:00 h to ensure data were comparable within and between sites (see Table S1 in Supporting Information).

The northernmost site is within the Abrolhos Bank, approximately 60 km off the coast of Bahia state, northeastern Brazil (17°58′S; 38°42′W), considered the largest and richest coral reefs in the South Atlantic (Francini-Filho et al. 2013). Three fringing reefs were sampled at the Abrolhos' Archipelago (Portinho Norte, Siriba and Mato Verde) and one additional reef at the top of coral pinnacles of the Parcel dos Abrolhos (Chapeirão), with depth varying from 3 to 10 m. All sampled areas are within the Abrolhos Marine National Park, established in 1983, but due to the inconsistent enforcement, there is occasional poaching in the areas (Francini-Filho & Moura 2008; Francini-Filho et al. 2013). The fringing reefs of the Archipelago are not massive coral formations and may be regarded as rocky reefs with a developing reef-building community (Francini-Filho et al. 2013). Benthic cover at the studied sites at Abrolhos was mainly epilithic algal community, coralline and fleshy algae, hydrocorals and scleractinian corals (see details in Appendix S1). Mean annual sea surface temperature at Abrolhos was 26.5°C, varying from 24° to 29°C and water temperature during fieldwork ranged from 27° to 29°C.

The southeastern study sites were subtropical rocky reefs located at the leeward side of the bay of Arraial do Cabo, Rio de Janeiro state (22°58′S; 41°59′W), southeastern Brazil. Three rocky reefs protected from winds and high waves, with depth varying between 3 and 11 m, were sampled: Anequim, Cardeiros, and Porcos (Fig.1). Despite having restrictions for fisheries since 1997, including a small no-take zone, the effectiveness of enforcement in these reefs is compromised. These reefs are composed of granite boulders ending in a sand bottom around 10 m (Ferreira, Peret & Coutinho 1998). Benthic cover was primarily epilithic algal community, sponges, zoanthids, and gorgonians (Appendix S1). This region is influenced by coastal upwelling events during the austral summer and spring; however, as the studied reefs are located in the leeward side of bays and inlets, this cold and nutrient-enriched water only bathes them for short periods and generally in deeper zones (Ferreira et al. 1998). Mean annual sea surface temperature at Arraial do Cabo was 23.5°C, varying from 20° to 27°C, and water temperature during fieldwork ranged from 23.5° to 25°C.

The southernmost study sites were subtropical rocky reefs of coastal islands in Santa Catarina state (27^°^36′S; 48^°^23′W), south Brazil, lying from 3 to 13 km from the coast and depths varying between 3 and 12 m (Fig.1). Four rocky reefs were studied: two with no protection from fisheries (Xavier and western Arvoredo) and two legally protected by the Arvoredo Marine Reserve since 2003 (eastern Arvoredo and Deserta), although with insufficient enforcement. These reefs are similar to the studied reefs in Arraial do Cabo, with granite boulders ending in sand bottoms around 10 m. Benthic cover was predominantly epilithic algal community and fleshy algae, in addition to sponges in deeper and zoanthids in shallower areas (Appendix S1). Mean annual sea surface temperature at Santa Catarina was 22.5°C, varying from 17° to 27°C, and water temperature during fieldwork ranged from 23° to 25°C. Although the mean temperatures between the southernmost and the southeastern sites were similar, 59% of the temperatures recorded between 1999 and 2009 at the southernmost site were below 23.5°C and 28% below 20°C, respectively, the annual mean and minimum temperatures of the southeastern site in the same period. Therefore, water temperature in the studied sites could be described as higher at Abrolhos (northernmost site), intermediate at Arraial do Cabo (southeastern site) and lower at Santa Catarina (southernmost site; Fig.1).

### Reef fish feeding pressure and abundance

Reef fish feeding pressure on the benthos was assessed with remote underwater video recordings. A video camera on a weighted tripod was placed on the reef substratum, and a 2-m transect tape was used to demarcate the recorded area and removed after 1 min. Each area was recorded for 15 min with the central 10 min of each video analyzed (sensu Longo & Floeter 2012). A minimum of three locations within each study site were sampled; an average of 15 replicated 2 m^2^ reef areas were haphazardly selected and video recorded (Table S1). This effort resulted in 290 video samples: 79 at Abrolhos, 90 at Arraial do Cabo, and 121 at Santa Catarina.

Each fish recorded feeding on the benthos was identified and assigned to a functional group; the total length estimated based on the transect tape initially deployed and the number of bites on the reef substratum were counted. A bite was considered every time a fish stroke the benthos with its jaws opened, closing its mouth subsequently, regardless of ingestion (Hoey & Bellwood 2009; Longo & Floeter 2012). Feeding pressure was determined by the total bites taken and body mass (kg) of each fish, to account for potential body size variation in the bite impact (Hoey & Bellwood 2009). The estimated biomass of fish was obtained from length–weight relationships from the literature (Froese & Pauly 2013). This method allowed fish feeding pressure to be evaluated from the perspective of several functional groups within different trophic categories, accounting for body size variation, per unit of time and area [(Bites × kg)/(2 m^2^ × 10 min)]. Here, feeding pressure is used as a metric of interaction strength sensu Paine (1992).

Fish density and biomass were estimated using 20 × 2 m strip transects (40 m^2^), where the diver swam identifying, counting, and estimating the size (total length) of larger (>5 cm) and shoaling fishes (Floeter et al. 2007). The fishes were assigned to functional groups, and the density and biomass of each species was obtained for each transect. These surveys were conducted at the same sites and period of day where video recording was taken and in the same or adjacent days to minimize differences in the assessed community. A total of 412 replicated transects were conducted: 148 at Abrolhos, 68 at Arraial do Cabo, and 200 at Santa Catarina.

### Functional groups

Despite potential problems associated with combining reef fishes into trophic and functional groups (Halpern & Floeter 2008), the functional perspective can provide a better understanding of ecosystems (e.g., Bellwood, Hughes & Hoey 2006; Hoey & Bellwood 2009). In this study, fishes were assembled into eight functional groups based on trophic categories and feeding behavior from the literature (e.g., Ferreira et al. 2004; Halpern & Floeter 2008; Green & Bellwood 2009; Longo & Floeter 2012), complemented by extensive field observations by the authors.

Particularly, for nominally herbivorous fishes (sensu Choat, Clements & Robbins 2004), we combined feeding modes and mobility, incorporating nutritional ecology into the discussions (Ferreira et al. 2004; Clements, Raubenheimer & Choat 2009). Herbivorous fishes were divided in two major categories: roving herbivores, comprising four functional groups according to their feeding mode, but intrinsic divergent nutritional ecology: scrapers, excavators, fine browsers, and rough browsers; and territorial herbivores (Ferreira et al. 2004). Scrapers and excavators can ingest a rich mass of detritus and animal matter trapped on the epilithic algal matrix and macroalgae they feed on and can occasionally predate live corals (Choat et al. 2004). However, scrapers remove less reef substratum than excavators, implying different contributions in reef bioerosion (Green & Bellwood 2009). Scrapers and excavators usually exhibit low ability for digesting algal carbohydrates, both endogenously and exogenously (Choat & Clements, 1998). As a result, they rely on protein-rich detritus to meet their nutritional requirements (Crossman, Choat & Clements 2005; Ferreira & Gonçalves 2006). Thus, while we are referring to scrapers and excavators simply as herbivores, in the Brazilian province, they possess similar proportions of plant material and sediment/detritus in their diets (Ferreira & Gonçalves 2006), and thus could be regarded as herbivorous–detritivorous species. In this study, scrapers included two acanthurids (surgeonfishes) and five scarine labrids parrotfishes, while excavator was comprised by a single species, the parrotfish *Scarus trispinosus* (following Ferreira & Gonçalves 2006; Francini-Filho & Moura 2008, Francini Filho et al. 2008; Longo & Floeter 2012). Other Brazilian parrotfish species (*Sparisoma amplum*) may act as excavators at larger sizes (Ferreira & Gonçalves 2006; Francini-Filho et al. 2008), but only small individuals of this species (<30 cm) were observed and thus classified as scrapers.

Browsers consistently feed on macroalgae by selecting and cropping individual algal components without removing the reef substratum or large amounts of detritus (Green & Bellwood 2009). Once they ingest primarily and almost exclusively macroalgae (i.e., algivorous), browser species can rely on endosymbiotic bacteria to ferment the highly complex algal carbohydrates they ingest (Choat & Clements, 1998). Here, browsers were also separated by feeding mode; those that crop small pieces of algae were labelled fine browsers (one acanthurid), whereas those that remove large pieces of algae (kyphosids) were labelled rough browsers. Apart from feeding rates, diet and isotopic niche (e.g., Lewis 1986; Ferreira & Gonçalves 2006; Dromard et al. 2014), very little is known about the nutrient assimilation of the fine browser species in this study, *Acanthurus coeruleus*, but it is likely to be an intermediate between those described to scrapers and browser (Choat & Clements, 1998).

Territorial herbivores, namely damselfishes, feed primarily on the epilithical algal matrix they farm within a defended territory, having a critical role in structuring benthic community through grazing and territoriality (Ceccarelli et al. 2001). The nutritional ecology of these fishes is poorly understood, but most species ingest large amounts of filamentous algae, animal material, and detritus (Wilson & Bellwood 1997; Ferreira et al. 1998). Thus, although territorial herbivores are likely to exhibit intermediate levels of gut fermentation between detritivores and herbivores (Choat et al. 2004), here, they were conservatively classified as a single group of territorial herbivores, comprising two damselfish species of the genus *Stegastes*.

The classification of fishes into the groups of mobile invertebrate feeders (i.e., feed on small benthic crustaceans, worms, molluscs), sessile invertebrate feeders (i.e., feed on cnidarians, molluscs, sponges), and omnivores (i.e., diversified diet including plankton, animal, and plant material) followed Ferreira et al. (2004). The nutritional ecology and physiology of omnivores can vary as a response to temperature (Behrens & Lafferty 2007) and although there might be a wide variation in feeding mode and mobility of reef fishes grouped as omnivores in this study (Ferreira et al. 2004), they were conservatively classified as a single group.

### Data analysis

A permutational multivariate analysis of variance (PERMANOVA; Anderson 2001) design was created based on the hierarchical sampling of localities (each reef) nested within sites (latitudes). Thus, “sites” were treated as a fixed factor and “locality” as random factors nested within “sites.” Such design was used to investigate both the variation in the intensity and composition of feeding pressure (response variables) across the sites and localities. The use of PERMANOVA on Euclidean Distance matrices calculated from only one variable yields an equivalent to Fisher's test, using permutations to calculate pseudo-F distributions and *P* -values (Anderson 2001). Thus, differences in the total feeding pressure (response variable) were investigated using this PERMANOVA design (999 iterations) on an Euclidean distance matrix obtained from square root transformed data. Similarly, this was also used to evaluate the total feeding pressure excluding the contribution of roving herbivores (response variable) and for the feeding impact of each functional group independently (response variables). Differences in the total nonmass-standardized bite rates (response variable) were also explored with and without roving herbivores to hold the consistency of the observed patterns for feeding pressure. Compositional changes in the feeding pressure of species and functional groups were assessed with the same PERMANOVA design (999 iterations), on Euclidean Distance matrices obtained from square root transformed data. This procedure was repeated excluding the contribution of roving herbivores for both species' and functional groups' matrices. Pairwise comparisons were conducted only when the fixed factor was significant. Such tests were performed in the software Primer 6 & PERMANOVA+ (Anderson & Gorley 2007).

To investigate how uniformly the feeding pressure was distributed among species and functional groups, an evenness measure was adapted from Hurlbert's (1971) probability of interspecific encounter. This index calculates the probability of two randomly sampled individuals from the assemblage representing different species, where 0 indicates that all individuals belong to the same species and 1 that all individuals differ. As applied in this study, it represents the probability of two randomly sampled units of feeding pressure within a pool being performed by different species or functional groups. This is measured in probability units and, different from other indices, is not prejudiced by sample size (Gotelli 2008). Cumulative rarefaction curves based on this index were performed with ECOSIM 7.0 (Gotelli & Entsminger 2007), providing confidence intervals for each curve through 1000 iterations. Thus, if the observed evenness and confidence intervals for a given site do not overlap the confidence interval generated to another site, the null hypothesis that the evenness of communities do not significantly differ can be rejected at *α * = 0.05 (Gotelli 2008). A flat pattern is expected in such rarefaction curves because of the index's independence to sample sizes (Gotelli 2008), but this approach was chosen over other methods because it standardizes the evenness measure to a common number of feeding pressure among sites and provides confidence intervals for hypothesis testing (Gotelli & Colwell 2001). The relationship between the mean feeding pressure, mean abundance, and biomass from visual censuses was assessed through Pearson's correlations, applied on log(x + 1) transformed data by species and functional groups pooled from all studied sites. For significant correlations, 95% confidence intervals were generated through 1000 iterations. Combining data from video recordings and visual censuses were possible because there is evidence of a limited difference in species detection between both techniques in the studied areas, with video recording having more advantages to assess feeding pressure and visual census to assess fish density (Longo & Floeter 2012). Combining mean fish biomass with the feeding pressure metric illustrates a compensation between bite rates and density of different sized individuals within and between species. Where more abundant but smaller individuals with higher bite rates could have a similar contribution to less abundant but bigger individuals with lower bite rates.

## Results

**Figure 2:**
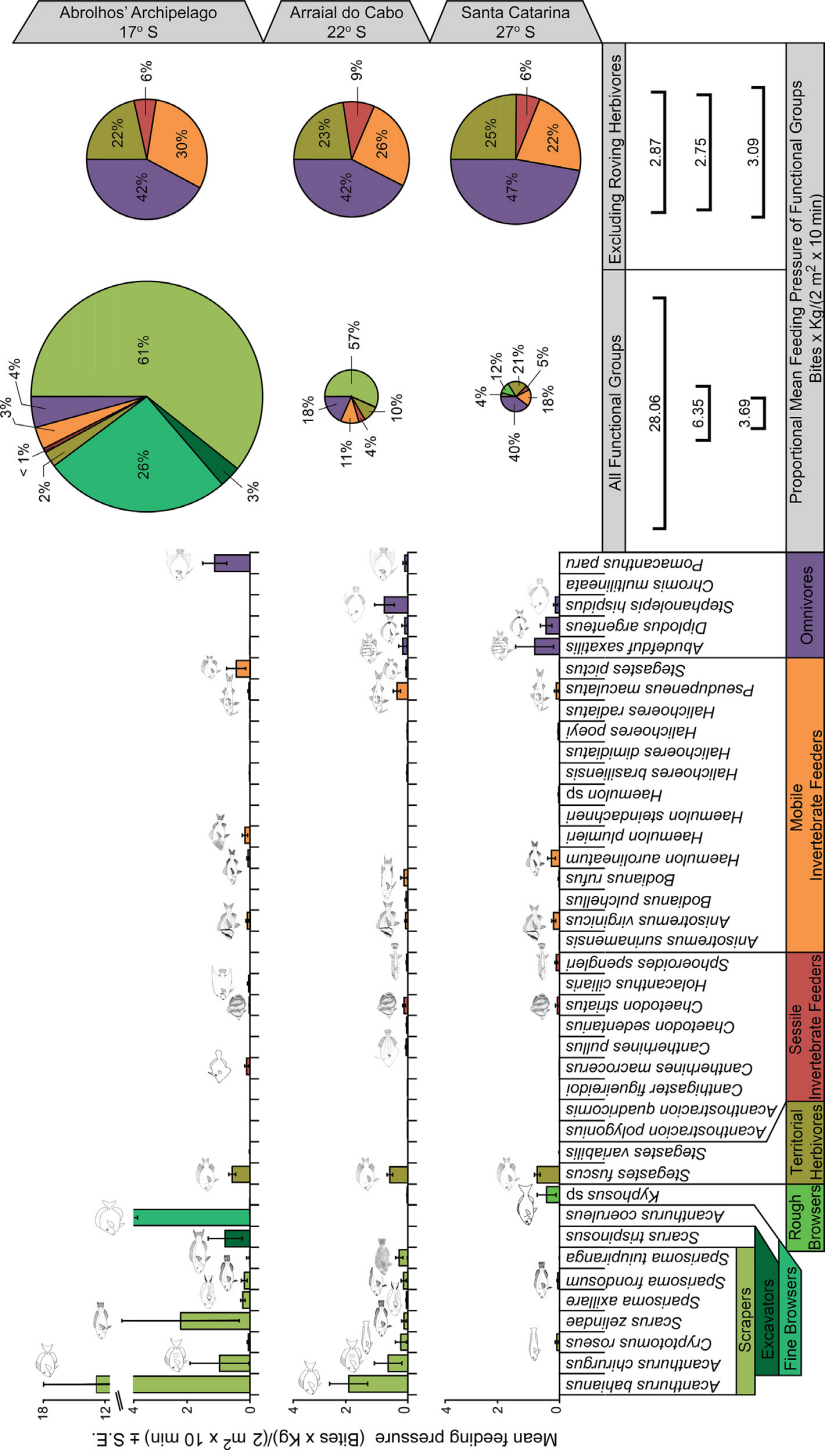
Mean feeding pressure of species (bar plots ± SE) and proportional mean feeding pressure of functional groups across the studied sites (pie charts). Circle sizes are proportional to the total feeding pressure.

There was a significant reduction in the total feeding pressure from the northern to the southernmost site (PERMANOVA, *P*  < 0·05; Fig.2), with values declining roughly ten times between them (Abrolhos = 28.06, Arraial do Cabo = 6.35, and Santa Catarina = 3.69; Table1). This was consistent with the decreasing pattern observed in the contribution of roving herbivores, particularly scrapers, whose feeding pressure significantly varied among the three sites (PERMANOVA; *P*  = 0.001; Table S2). However, total feeding pressure did not vary among sites when all roving herbivores were excluded from the analysis (PERMANOVA; *P*  = 0.001; Table1). Similarly, non-mass-standardized total bite rates followed the same pattern with and without roving herbivores (see Fig. S1 in Supporting Information; Table S3).

**Table 1:** Summary of permutational multivariate analysis of variance (PERMANOVA) for total feeding pressure of all functional groups and excluding roving herbivores, with site as a fixed factor and locality as a random factor nested within sites

Variable	Source of variation	df	MS	Pseudo-F	*P* -value
Total feeding pressure of the entire community					
Main test	Site	2	14.585	8.454	**0.014**
	Locality (Site)	8	1.756	1.422	0.181
Pairwise comparisons	t	*P* -value			
*Abrolhos* vs. *Arraial*	2.069	0.099			
*Abrolhos* vs. *Santa Catarina*	4.556	**0.018**			
*Arraial* vs. *Santa Catarina*	1.897	0.078			
Total feeding pressure excluding roving herbivores					
						Main test	Site	2	0.445	0.309	0.729
							Locality (Site)	8	1.492	2.412	**0.016**

df, degrees of freedom; MS, mean squares

Pairwise comparisons are only provided for the fixed factor. Pseudo-F distribution and *P* -values obtained through 999 iterations. Significant differences are presented in bold (*P*  < 0.05).

Scrapers were the most representative functional group at the northernmost and southeastern sites contributing 61% and 57% of the total feeding pressure and accounting for approximately 70% and 98% of the feeding pressure exerted by roving herbivores, respectively. The feeding pressure of this group at the southernmost site was 4% of the total. Excavators and fine browsers were only recorded at the northernmost reefs (Abrolhos) while rough browsers were rarely observed along the three studied sites, even though it was the most notable roving herbivore in the southernmost reefs (Santa Catarina). Omnivores, in turn, presented an inverse pattern, acting as the main group in the southernmost site with 40% of the feeding pressure, decreasing to 18% at the southeastern and 4% at the northernmost sites. The relative functional contribution of scrapers decreased around 15 times from the northern to the southernmost site while omnivores' increased 10 times (Fig.2). Feeding pressure of territorial herbivores and mobile invertebrate feeders also increased toward the southernmost site, being, respectively, 2% and 3% at Abrolhos and 21% and 18% in Santa Catarina, while sessile invertebrate feeders were always below 5%.

Most feeding pressure at the northernmost site (Abrolhos) was performed by acanthurid species of two functional groups (the scraper *Acanthurus bahianus* and the fine browser *A. coeruleus*) and at the southeastern site (Arraial do Cabo) by two scraper acanthurid species (*A. bahianus* and *A. chirurgus*). In the southernmost study area (Santa Catarina), the rough browser *Kyphosus* sp. was the roving herbivore performing higher feeding pressure, while the territorial herbivore *Stegastes fuscus* and the omnivorous species *Abudefduf saxatilis* were the major contributors (Fig.2).

The composition of species and functional groups performing feeding pressure significantly varied among studied sites (PERMANOVA, *P*  < 0.05; Table2). However, excluding all roving herbivores, the composition of functional groups did not vary among the sites but species within these groups did (Fig.2; Table2). Feeding pressure was more evenly distributed among the functional groups in the southernmost site (Table3; Fig. S2A), with an evenness of 0.75, followed by the southeastern site, with 0.63, and the northernmost site, with 0.56. A similar pattern was observed in the species level; Abrolhos displayed a lower evenness (0.70) in comparison to Arraial do Cabo (0.85) and Santa Catarina (0.86) in the presence of all roving herbivores (Fig. S2C). However, when all roving herbivores were excluded from both analyses, the evenness of feeding pressure did not vary among the study sites (Table3; Fig. S2B and D).

**Table 2:** Summary of permutational multivariate analysis of variance (PERMANOVA) and pairwise comparisons on the composition of feeding pressure, with site as a fixed factor and locality as a random factor nested within sites

Variables	Source of Variation	df	MS	Pseudo-F	*P* -value
Entire community					
Functional groups					
Main test	Sites	2	90.081	7.678	**0.002**
	Locality (Site)	8	11.889	1.317	0.136
Pairwise comparison (Site)	t	*P*			
*Abrolhos* vs. *Arraial*	2.291	**0.006**			
*Abrolhos* vs. *Santa Catarina*	2.209	**0.033**			
*Arraial* vs. *Santa Catarina*	3.499	**0.008**			
Species					
Main test	Sites	2	77.687	5.919	**0.002**
	Locality (Site)	8	13.316	1.351	0.087
Pairwise comparison (Site)	t	*P*			
*Abrolhos* vs. *Arraial*	2.077	**0.036**			
*Abrolhos* vs. *Santa Catarina*	3.066	**0.006**			
*Arraial* vs. *Santa Catarina*	2.024	**0.021**			
Excluding roving herbivores					
Functional groups					
Main test	Sites	2	1.175	0.169	0.994
	Locality (Site)	8	7.226	3.354	**0.001**
Species					
Main test	Sites	2	11.516	2.029	**0.016**
	Locality (Site)	9	5.860	2.370	**0.001**
Pairwise comparison (Site)	t	*P*			
*Abrolhos* vs. *Arraial*	1.451	**0.048**			
*Abrolhos* vs. *Santa Catarina*	1.434	**0.052**			
*Arraial* vs. *Santa Catarina*	1.469	0.097			

df, degrees of freedom; MS, mean squares.

Pairwise comparisons are only provided for the fixed factor. Pseudo-F distribution and *P* -values obtained through 999 iterations. Significant differences are presented in bold (*P*  < 0.05).

**Table 3:** Evenness (Hurlbert's PIE) of feeding pressure within functional groups and species, with and without roving herbivores, and comparisons among the three sites

Variables	Abrolhos (17°S)	Arraial do Cabo (22°S)	Santa Catarina (27°S)	Comparison (95% CI)
Functional groups				
All	**0.559**	**0.625**	**0.749**	**AB=AC≠SC**
Except Roving herbivores	0.682	0.696	0.665	AB=AC=SC
Species				
All	**0.704**	**0.849**	**0.861**	**AB≠AC=SC**
Except Roving herbivores	0.746	0.835	0.823	AB=AC=SC

The evenness resulted from cumulative rarefaction curves generated for each site and comparisons are based on the 95% confidence intervals (1000 iterations; Figure S2). Significant differences are presented in bold.

**Figure 3:**
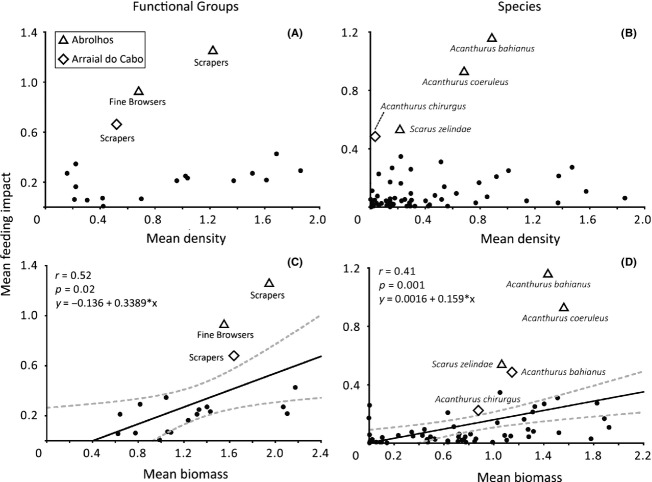
Pearson's correlation between mean feeding pressure, mean density, and biomass of functional groups (A, C) and species (B,D). Data from the three study sites were pooled in this analysis and log(x + 1) transformed. Triangles indicate data from Abrolhos and diamonds from Arraial do Cabo. Only herbivore's functional groups and species identified as critical are indicated. Gray dashed lines indicate 95% confidence intervals generated through 1000 iterations.

Feeding pressure was not correlated to either abundance of functional groups (*r*  = 0.22, *P*  = 0.37) or species (*r*  = 0.20, *P*  = 0.11, Fig.3A and B). However, there was a significant and positive correlation between feeding pressure and biomass of both functional groups (*r*  = 0.52, *P*  = 0.02; Fig.3C) and species (*r*  = 0.41, *P*  = 0.001; Fig.3D). Two functional groups presented a higher feeding pressure than predicted by their biomass: scrapers at Abrolhos and Arraial do Cabo, and fine browsers at Abrolhos (Fig. 3C). Also, the feeding pressure of all acanthurid species was disproportionate to their biomass at Abrolhos and Arraial do Cabo (Fig.3D). The territorial herbivore *Stegastes fuscus*, the mobile invertebrate feeder *S. pictus*, and the omnivore *Pomacanthus paru* at Abrolhos in addition to the omnivore *Stephanolepis hispidus* at Arraial do Cabo also performed a higher feeding pressure than predicted by their biomass.

## Discussion

The decline in the total feeding pressure (pooling all trophic categories) from the northern to the southernmost sites was mostly driven by the reduction in the feeding pressure of roving herbivores whose richness and abundance decline beyond the latitude 23°S (i.e., Arraial do Cabo) in the western Atlantic (Ferreira et al. 2004; Floeter et al. 2005). The feeding pressure of roving herbivores also determined changes in the composition and distribution of the feeding pressure among the studied areas.

The distinct patterns of feeding pressure between roving herbivores and other trophic categories suggest that an herbivory-related constraint could be taking place. The potential physiological constraints on tropical herbivorous fishes (e.g., acanthurids, scarids) to digest and assimilate plant material in lower temperatures (Gaines & Lubchenco 1982) have already been used as an explanatory hypothesis for declining bite rates, richness, and abundance of herbivorous fish in the South Atlantic (Ferreira et al. 2004; Floeter et al. 2005). Although the difference in the mean sea surface temperature between the southeastern and the southernmost sites was 1°C, the minimum temperature varied 3°C between the areas (20° and 17°C, respectively). Additionally, temperatures below 20°C, a range that can affect the distribution and bite rates of herbivorous fishes (Floeter et al. 2005), were only recorded at the southernmost site. Thus, fish could be responding more to long-term temperature patterns than to the instantaneous temperature (see Bennett and Bellwood 2011). Because most roving herbivores in the present study (scrapers) are in fact herbivorous–detritivorous and rely on a higher protein ingestion from detritus, associated microbiota and benthic invertebrates (Choat et al. 2004; Ferreira & Gonçalves 2006; Dromard et al. 2014), the herbivory-related constraint related to this functional group should be reconsidered. Additionally, it is still poorly understood how the relative contribution of detritus, invertebrates, and plant material in the diet of these species may vary across geographic scales. Hence, directly assigning the observed declining feeding pressure to constraints in their digestive ability or only to temperature would be inaccurate.

A recent study comparing demographic traits (e.g., growth rates, life span) and abundance of two reef fish species with distinct nutritional ecology (herbivore vs. carnivore) found a consistent pattern for both species along a temperature gradient despite their different feeding strategies (Trip et al. 2013). However, the herbivorous fish in that study, *Odax pullus*, consistently feeds on macroalgae (i.e. *,* algivore) and belong to a temperate related clade, being distributed among subtropical and temperate reefs of New Zealand and Australia (Clements et al. 2004). Thus, the lower temperatures do not constrain their ability to digest and assimilate plant material as it could potentially do to tropical originated herbivorous fish that inhabit warmer habitats and also rely on detritus in their diet (e.g., scarinis and some acanthurids). Studies applying consistent methods across a large spatial scales, comprising tropical and subtropical originated herbivorous fishes, with distinct nutritional ecology, are needed before a precise conclusion may be drawn on this matter.

Alternatively, characteristics of the reefs, rates of primary production, algae biomass, chemical defenses, and nutritional quality of algae among the studied areas could also be important explanatory factors for the feeding pressure patterns of roving herbivores (Hay 1997; Cebrian et al. 2009; Poore et al. 2012). The reefs at the northernmost area, for example, present more tropical characteristics in comparison to the other studied sites, comprising twenty species of scleractinian corals and higher coral cover (Leão, Kikuchi & Testa 2003; Appendix S1). Conversely, the rocky reefs of the southeastern and southernmost areas comprise fewer coral species (five and two, respectively; Leão et al. 2003) and are more similar in terms of reef composition (e.g., granite boulders with low coral cover; Appendix S1; see pictures in Fig.1) and trophic structure of reef fish assemblages (Ferreira et al. 2004). Thus, one could expect that the feeding pressure of roving herbivores would be higher at the northernmost site but similar between the two other areas. However, there was an abrupt decline in the feeding pressure of this group between the southeastern to the southernmost areas, coinciding with the decline in the abundance of roving herbivores in the same areas, primarily attributed to temperature-related factors (Ferreira et al. 2004; Floeter et al. 2005). Differences in the macroalgal availability are also unlikely to be the explanatory factor to the patterns in the present study because: (1) macroalgal cover did not significantly vary between the three studied sites (Appendix S1); (2) the richness of macroalgae did not decline from the northern to the southernmost studied sites; and (3) when the macroalgal composition by genera and species was compared along the Brazilian coast, the northernmost site was more similar to tropical areas, while the southeastern and southernmost sites belonged to the warm temperate group (Horta et al. 2001).

Macroalgal removal by reef fishes at the Great Barrier Reef declined ten times between the northern and southernmost sites across a 7° latitudinal gradient, pooling macroalgae browsers and scrapers (Bennett and Bellwood 2011). This was consistent with the 10-fold reduction in herbivory across the three sites spanning 10° of latitude in the present study, also pooling browsers, scrapers, and excavators. Another common outcome was the dominance of a few species comprising most of macroalgal removal (browsers and scrapers) and feeding pressure (mainly scrapers), generating low functional redundancy.

The dominance of all roving herbivores combined, also affected the composition and distribution of feeding pressure among different trophic categories. The evenness pattern of trophic interactions within functional groups and species was determined by a few strong (scrapers) and several weak interactions (territorial herbivores, mobile invertebrate feeders, and omnivores). Manipulation of grazing by invertebrates in an intertidal habitat also revealed a skewed distribution of trophic interactions toward dominant species (Paine 1992). In kelp forests, the combination of weak interactions from different species has important effects on food webs (Sala & Graham 2002). If the dominance of trophic interactions by few species can result in less stability (sensu Duffy 2002), the higher evenness of trophic interactions observed in the southernmost site could translate into a higher functional redundancy of trophic links and would provide more stability and resistance to the loss of biodiversity (Duffy 2002).

The incongruence between feeding pressure and density of species and functional groups can be reflecting the fact that fish from different trophic groups and nutritional strategies present different bite rates (Choat, Clements & Robbins 2004). However, from a general perspective, it may be also interpreted in the notion that species performing more function than would be predicted based on their biomass have the potential to be critical species to ecosystems (Power et al. 1996). Even though we did not assess the amount of removed substratum and the effects of fish feeding pressure on the benthos, scrapers and fine browsers adding up to three *Acanthurus* and a *Scarus* species (Fig.3) can be suggested as critical because: (1) they performed most of the total feeding pressure; (2) their contribution was greater than would be predicted based on their biomass (Power et al. 1996); (3) their feeding pressure and its consequent benthic biomass removal can be critical ecosystem processes in reef systems (e.g., Lewis 1986; Hoey & Bellwood 2009); and (4) these functional groups and mainly acanthurid species determined the large-scale spatial variation in fish feeding pressure.

There is a widespread notion, mostly associated to herbivores and top predators, that the removal of critical species and functional groups by overfishing may strongly impact the functioning of reefs often resulting in phase shifts (Mumby 2006; Hoey & Bellwood 2009; Estes et al. 2011; Cheal et al. 2013). Despite being the most species rich functional group in reef systems, invertebrate feeders and their relative contribution to ecosystem function are poorly discussed and seldom regarded as critical (Jones, Ferrel & Sale 1991). Omnivores are also commonly overlooked, especially considering that this category often comprises species with different feeding modes that can perform important functional roles in the reefs. While the consequences of loosing these groups are still to be understood, fishing pressure is increasingly affecting different trophic levels including herbivores, invertebrate feeders, and omnivores (Floeter, Halpern & Ferreira 2006; Estes et al. 2011; Bender et al. 2013).

On a large scale, it is unlikely that fishing pressure drove the decline in the feeding pressure of roving herbivores because the exploitation of this group does not occur in the southernmost studied area where they presented the lower feeding pressure, but it is intense between the northeastern coast of Brazil and the southeastern studied sites where they comprised most of the feeding pressure (see Floeter et al. 2006; Nóbrega & Lessa 2007; Cunha et al. 2012; Bender et al. 2014). Thus, if the differences in the fishing pressure were the main drivers of the observed large-scale patterns, a completely opposite scenario would be expected, where roving herbivores would be more important in the southernmost reefs. Conversely, on a local scale, the patterns of feeding pressure are likely influenced by the decline or disappearance of large herbivores. For example, large-bodied scarids, such as the green beak parrotfish *Scarus trispinosus*, have been historically under strong fishing pressure at Abrolhos and Arraial do Cabo (Floeter et al. 2006; Bender et al. 2013, 2014). This species was recently categorized as endangered in the IUCN red list given its 50% population decline over the past 20–30 years caused by overfishing (Ferreira et al. 2012; IUCN 2012). At the southeastern site (Arraial do Cabo), both local ecological knowledge and underwater visual census data show an historical decline in this species populations (Bender et al. 2014). Thus, the absence of feeding pressure by *S. trispinosus* at Arraial do Cabo (Fig.2) probably results from overfishing limiting species functional roles, while their feeding pressure at Abrolhos could have been even higher in the past. Acanthurids identified as critical at the reefs of Abrolhos and Arraial Cabo are also under fishing pressure in the northeastern Brazilian coast (Nóbrega & Lessa 2007; Cunha et al. 2012), which could be affecting their functional roles in the reefs. Invertebrate feeders (e.g., *Pseudupeneus maculatus*) and omnivores (e.g., *Diplodus argenteus*) also demand urgent attention because both are under fishing pressure in the Brazilian reefs (Floeter et al. 2006; Cunha et al. 2012), and the consequences of their potential ecological extinction are difficult to predict. Even though some of these species do not fit the criteria to be in most red lists, their ecological roles are threatened and must be protected.

The remarkable changes in the intensity and composition of fish feeding pressure on the benthos across the studied sites were driven by the declining contribution of roving herbivores. Comparing how species with different feeding ecology affect the strength and distribution of trophic interactions across large spatial scales can shed light to new interaction-based approaches to functional redundancy and conservation of reef ecosystems.

## Supporting Information

Appendix S1 Information on the benthic cover at the studied sites.

**Table S1.** Sample summary of the field effort at the study sites. **Table S2.** Summary of PERMANOVA of total feeding pressure per functional group between the studied sites. **Table S3.** Summary of PERMANOVA for total non-mass-standardized bite rates between the studied sites, with and without roving herbivores.

Figure S1 Mean feeding pressure and non-mass-standardized bite rates between the studied sites.

Figure S2 Cumulative rarefaction curves of the evenness of feeding pressure within functional groups and species, with and without roving herbivores, and comparisons between the studied sites.
