# Supplementary material for: Herbivory drives large-scale spatial variation in reef fish trophic interactions
Source: Ecol Evol. 2014 Nov 22;4(23):4553–66. doi: 10.1002/ece3.1310 (PMC4264904; doi:10.1002/ece3.1310)
Supplement: Appendix S1 — Information on the benthic cover at the studied sites. [file ece30004-4553-sd1.docx]

**Ecology and Evolution**

**Appendix S1: Information on the benthic cover at the studied sites**

Benthic cover at the studied sites was assessed through sets of five photoquadrats taken inside the recorded areas right after the recording period. A total of 78 areas were surveyed at Abrolhos (Chapeirão= 15; Mato Verde= 7; Portinho Norte= 40; Siriba=16), 86 at Arraial do Cabo (Anequim= 27; Cardeiros= 35; Porcos= 24) and 33 at Santa Catarina (Arvoredo West= 17; Xavier=16). All images were analysed in the software CPCE version 4.1 (Kohler & Gill 2006), randomly positioning points over the images and identifying benthic organisms below each of them. Organisms were classified in seven broad categories based on the dominant groups: macroalgae, coralline crustose algae, scleractinian corals, other anthozoans, sponges and others. Percent cover of each benthic group (relative abundance) was obtained for each set of five images (sample) and averaged to describe the sites. Differences in the overall benthic cover composition (response variable) were investigated through permutational multivariate analysis of variance (PERMANOVA; Anderson 2001), according to our hierarchical sampling of localities (each reef) nested within sites (latitudes). Thus, “sites” were treated as a fixed factor and “locality” as a random factor nested within “sites”. Differences in the percent cover of each benthic group were also assessed through the same PERMANOVA design, but applied on each benthic group separately. All benthic cover data was transformed through the arc sine of the square root and PERMANOVA tests were performed on Euclidian Distance matrices using the software Primer 6 & PERMANOVA+ (Anderson & Gorley 2007). The use of PERMANOVA on Euclidean Distance matrix obtained for one single variable yields an equivalent to Fisher’s test using permutations and generates pseudo-F distribution and p-values (Anderson 2001). The category “others” was excluded from the analysis because it lacks biological meaning, once organisms grouped in this category are not necessarily the same in the three sites. The overall benthic cover significantly varied between sites and localities within sites (Table AS1 and AS2). Macroalgal cover did not significantly vary between the sites, but the cover of scleractinian corals, sponges and sand did vary (Table AS3).

**Table AS1.** Mean percent cover (± S.E.) of benthic groups in each site. Percent cover values that varied between sites are displayed in bold. * Detailed results of the test on differences between sites are presented in Table AS3.

| **Benthic groups** | **Sites** | | | **Difference between sites*** |
| --- | --- | --- | --- | --- |
|  | **Abrolhos**  **(17^o^S)** | **Arraial do Cabo**  **(22^o^S)** | **Santa Catarina**  **(27^o^S)** |  |
| **Macroalgae** | 61.53%  (±2.44) | 53.49%  (±2.18) | 59.23%  (±2.75) | n.s. |
| **Crustose coralline algae** | 11.64%  (±1.00) | 7.43%  (±0.75) | 20.63%  (±1.92) | n.s. |
| **Scleractinian Corals** | **11.29%**  (±1.28) | **2.63%**  (±0.45) | **0.00**  (±0.00) | AB ≠ AC |
|  |  |  |  | AB ≠ SC |
|  |  |  |  | AC = SC |
| **Other Anthozoans** | 6.29%  (±1.44) | 18.14%  (±2.27) | 0.90  (±0.42) | n.s. |
| **Sponges** | **1.23%**  (±0.19) | **3.92%**  (±0.82) | **0.82**  (±0.31) | AB ≠ AC |
|  |  |  |  | AB = SC |
|  |  |  |  | AC = SC |
| **Sand** | **2.55%**  (±0.60) | **10.48%**  (±1.16) | **13.28**  (±1.92) | AB ≠ AC |
|  |  |  |  | AB ≠ SC |
|  |  |  |  | AC = SC |
| **Others** | 5.85%  (±1.06) | 6.63%  (±1.09) | 5.27  (±1.47) | not tested |

**Table AS2.** Summary of permutational multivariate analysis of variance (PERMANOVA) for the overall benthic cover composition, with site as a fixed factor and locality as a random factor nested within sites. Pairwise comparisons are only provided for the fixed factor. PERMANOVA was applied on an Euclidean Distance matrix obtained from arc sine of the square root transformed data. Pseudo-F distribution and p-values obtained through 999 iterations. Significant differences are presented in bold (p < 0.05). df = degrees of freedom; MS = mean squares.

| **Variable** | **Source of variation** | **df** | **MS** | **Pseudo-F** | ***p*-value** |
| --- | --- | --- | --- | --- | --- |
| **Overall benthic cover composition** |  |  |  |  |  |
| **Main Test** | Site | 2 | 3.55 | 2.893 | **0.031** |
|  | Locality (Site) | 6 | 1.36 | 8.997 | **0.001** |
| **Pairwise comparisons** | **t** | ***p*-value** |  |  |  |
| *Abrolhos vs. Arraial* | 1.561 | 0.094 |  |  |  |
| *Abrolhos vs. Santa Catarina* | 2.039 | **0.015** |  |  |  |
| *Arraial vs. Santa Catarina* | 0.168 | 0.108 |  |  |  |

**Table AS3.** Summary of permutational multivariate analysis of variance (PERMANOVA) for each benthic group (response variable) with site as a fixed factor and locality as a random factor nested within sites. Pairwise comparisons are only provided for the fixed factor. The test was applied on Euclidean Distance matrices obtained from arc sine of square root transformed data (999 iterations). Significant differences are presented in bold (p < 0.05). df = degrees of freedom; MS = mean squares.

| **Functional Group** | **Source of variation** | | **df** | | **MS** | | **Pseudo-F** | | ***p*-value** | |
| --- | --- | --- | --- | --- | --- | --- | --- | --- | --- | --- |
| **Macroalgae** |  | |  | |  | |  | |  | |
|  | Site | | 2 | | 0.005 | | 0.113 | | 0.881 | |
|  | Locality (Site) | | 6 | | 0.540 | | 16.594 | | **0.001** | |
| **Crustose Coralline Algae** | **Source of variation** | **df** | | **MS** | | **Pseudo-F** | | ***p*-value** | |  |
|  | Site | | 2 | | 0.572 | | 5.385 | | 0.071 | |
|  | Locality (Site) | | 6 | | 0.117 | | 7.523 | | **0.001** | |
| **Scleractinian Corals** | **Source of variation** | | **df** | | **MS** | | **Pseudo-F** | | ***p*-value** | |
|  | Site | | 2 | | 0.838 | | 7.948 | | **0.016** | |
|  | Locality (Site) | | 6 | | 0.116 | | 5.900 | | **0.001** | |
| **Pairwise comparisons** | **t** | | ***p*-value** | |  | |  | |  | |
| *Abrolhos vs. Arraial* | 2.550 | | **0.045** | |  | |  | |  | |
| *Abrolhos vs. Santa Catarina* | 3.765 | | **0.017** | |  | |  | |  | |
| *Arraial vs. Santa Catarina* | 2.273 | | 0.113 | |  | |  | |  | |
| **Other Anthozoans** | **Source of variation** | | **Df** | | **MS** | | **Pseudo-F** | | ***p*-value** | |
|  | Site | | 2 | | 1.083 | | 2.702 | | 0.158 | |
|  | Locality (Site) | | 6 | | 0.444 | | 8.887 | | **0.001** | |
| **Sponges** | **Source of variation** | | **df** | | **MS** | | **Pseudo-F** | | ***p*-value** | |
|  | Site | | 2 | | 0.188 | | 12.802 | | **0.007** | |
|  | Locality (Site) | | 6 | | 0.016 | | 2.689 | | **0.017** | |
| **Pairwise comparisons** | **t** | | ***p*-value** | |  | |  | |  | |
| *Abrolhos vs. Arraial* | 3.671 | | **0.021** | |  | |  | |  | |
| *Abrolhos vs. Santa Catarina* | 1.801 | | 0.194 | |  | |  | |  | |
| *Arraial vs. Santa Catarina* | 3.419 | | 0.089 | |  | |  | |  | |
| **Sand** | **Source of variation** | | **df** | | **MS** | | **Pseudo-F** | | ***p*-value** | |
| **Main Test** | Site | | 2 | | 0.816 | | 7.047 | | **0.043** | |
|  | Locality (Site) | | 6 | | 0.127 | | 4.599 | | **0.001** | |
| **Pairwise comparisons** | **t** | | ***p*-value** | |  | |  | |  | |
| *Abrolhos vs. Arraial* | 3.381 | | **0.011** | |  | |  | |  | |
| *Abrolhos vs. Santa Catarina* | 4.359 | | **0.046** | |  | |  | |  | |
| *Arraial vs. Santa Catarina* | 0.617 | | 0.594 | |  | |  | |  | |

**Literature cited**

Anderson, M.J. (2001) A new method for non-parametric multivariate analysis of variance. *Austral Ecology*, **26**, 32–46.

Anderson, M. J. & Gorley, R. N. (2007) PERMANOVA+ for primer: guide to statistical methods. PRIMER-E, Plymouth.

Kohler, K.E., Gill, S.M. (2006) Coral Point Count with Excel extensions (CPCe): A Visual Basic program for the determination of coral and substrate coverage using random point count methodology. *Computers and Geosciences*, **32(9)**, 1259–1269.
