# Supplementary material for: Herbivory drives large-scale spatial variation in reef fish trophic interactions
Source: Ecol Evol. 2014 Nov 22;4(23):4553–66. doi: 10.1002/ece3.1310 (PMC4264904; doi:10.1002/ece3.1310)
Supplement: Supplementary file 2 — Table S1. Sample summary of the field effort at the study sites. Table S2. Summary of PERMANOVA of total feeding pressure per functional group between the studied sites. Table S3. Summary of PERMANOVA for total non-mass-standardized bite rates between the studied sites, with and without roving herbivores. [file ece30004-4553-sd2.docx]

**Supporting Information**

**Table S1.** Sample summary of the field effort at the study sites along the Brazilian Coast. UVC= underwater visual census.

| **Site** | **Abbreviation** | **Coordinates** | **Depth** | **N**  **(Videos)** | **N**  **(UVC)** | **Month** | **Year** |
| --- | --- | --- | --- | --- | --- | --- | --- |
| **Abrolhos (17° S)** |  |  |  |  |  |  |  |
| Chapeirão | CHA | 17^o^58’38.62’’ S  38^o^43’19.40’’ W | 6-12m | 15 | 28 | March | 2010 |
| Mato Verde | MTV | 17^o^57’53.78’’ S  38^o^42’04.69’’ W | 4-10m | 8 | 25 | March | 2010 |
| Portinho Norte | PTN | 17^o^57’43.52’’ S  38^o^41’52.51’’ W | 3-10m | 40 | 50 | March | 2010 |
| Siriba | SRB | 17^o^58’10.63’’ S  38^o^42’38.19’’ W | 4-10m | 16 | 45 | March | 2010 |
| Sum of samples |  |  |  | **79** | **148** |  |  |
| **Arraial do Cabo (22**° **S)** |  |  |  |  |  |  |  |
| Anequim | ANE | 22^o^58’51.81’’ S  41^o^59’03.34’’ W | 3-12m | 30 | 20 | April | 2011 |
| Cardeiros | CAR | 22^o^57’55.27’’ S  42^o^00’06.34’’ W | 3-12m | 30 | 20 | April | 2011 |
| Porcos | POR | 22^o^58’03.27’’ S  41^o^59’39.11’’W | 3-12m | 30 | 20 | April | 2011 |
| Sum of samples |  |  |  | **90** | **64** |  |  |
| **Santa Catarina (27**° **S)** |  |  |  |  |  |  |  |
| Eastern Arvoredo | ARV_E | 27^o^17’34.15’’ S  48^o^21’27.66’’ W | 3-12m | 25 | 25 | Feb-Apr | 2011 |
| Western Arvoredo | ARV_W | 27^o^17’06.23’’ S  48^o^22’17.16’’ W | 3-12m | 33 | 51 | Feb-Apr | 2010/2011 |
| Deserta | DES | 27^o^16’09.23’’ S  48^o^19’49.13’’ W | 3-12m | 33 | 44 | Feb-Apr | 2011 |
| Xavier | XAV | 27^o^36’32.35’’ S  48^o^23’09.27’’ W | 3-12m | 30 | 80 | Feb-Apr | 2010/2011 |
| Sum of samples |  |  |  | **121** | **200** |  |  |
|  |  |  |  |  |  |  |  |
| **Total of samples** |  |  |  | **290** | **412** |  |  |

**Table S2.** Summary of permutational multivariate analysis of variance (PERMANOVA) for total feeding pressure of each functional group with site as a fixed factor and locality as a random factor nested within sites. Pairwise comparisons are only provided for the fixed factor. PERMANOVA was applied on Log (x+1) transformed data and using Euclidean Distance and thus yields an equivalent to Fisher’s test using permutations (Anderson 2001). Pseudo-F distribution and p-values obtained through 999 iterations. Significant differences are presented in bold (p < 0.05). df = degrees of freedom; MS = mean squares.

| **Functional Group** | **Source of variation** | | **df** | | **MS** | | **Pseudo-F** | | ***p*-value** | |
| --- | --- | --- | --- | --- | --- | --- | --- | --- | --- | --- |
| **Scrapers** |  | |  | |  | |  | |  | |
| **Main Test** | Site | | 2 | | 18.899 | | 33.498 | | **0.001** | |
|  | Locality (Site) | | 9 | | 0.436 | | 0.407 | | 0.930 | |
| **Pairwise comparisons** | **t** | | ***p*-value** | |  | |  | |  | |
| *Abrolhos vs. Arraial* | 11.555 | | **0.007** | |  | |  | |  | |
| *Abrolhos vs. Santa Catarina* | 2.478 | | **0.031** | |  | |  | |  | |
| *Arraial vs. Santa Catarina* | 5.869 | | **0.004** | |  | |  | |  | |
| **Territorial herbivores** | **Source of variation** | **df** | | **MS** | | **Pseudo-F** | | ***p*-value** | |  |
| **Main Test** | Site | | 2 | | 0.122 | | 0.231 | | 0.729 | |
|  | Locality (Site) | | 9 | | 0.607 | | 2.820 | | **0.003** | |
| **Sessile Invertebrate Feeders** | **Source of variation** | | **df** | | **MS** | | **Pseudo-F** | | ***p*-value** | |
| **Main Test** | Site | | 2 | | 0.772 | | 1.669 | | 0.219 | |
|  | Locality (Site) | | 9 | | 0.558 | | 6.731 | | **0.003** | |
| **Mobile Invertebrate Feeders** | **Source of variation** | | **df** | | **MS** | | **Pseudo-F** | | ***p*-value** | |
| **Main Test** | Site | | 2 | | 0.849 | | 0.123 | | 0.890 | |
|  | Locality (Site) | | 9 | | 0.786 | | 2.502 | | **0.027** | |
| **Omnivores** | **Source of variation** | | **df** | | **MS** | | **Pseudo-F** | | ***p*-value** | |
| **Main Test** | Site | | 2 | | 1.731 | | 0.940 | | 0.463 | |
|  | Locality (Site) | | 9 | | 2.183 | | 4.467 | | **0.003** | |

**Table S3.** Summary of permutational multivariate analysis of variance (PERMANOVA) for total non-mass-standardized bite rate of all functional groups and excluding roving herbivores, with site as a fixed factor and locality as a random factor nested within sites. Pairwise comparisons are only provided for the fixed factor. Pseudo-F distribution and p-values obtained through 999 iterations. Significant differences are presented in bold (p < 0.05). df = degrees of freedom; MS = mean squares.

| **Variable** | **Source of variation** | **df** | **MS** | **Pseudo-F** | ***p*-value** |
| --- | --- | --- | --- | --- | --- |
| **Total bite rate of the entire community** |  |  |  |  |  |
| **Main Test** | Site | 2 | 810.05 | 10.479 | **0.014** |
|  | Locality (Site) | 8 | 80.61 | 3.295 | 0.181 |
| **Pairwise comparisons** | **t** | ***p*-value** |  |  |  |
| *Abrolhos vs. Arraial* | 2.203 | 0.062 |  |  |  |
| *Abrolhos vs. Santa Catarina* | 4.592 | **0.003** |  |  |  |
| *Arraial vs. Santa Catarina* | 2.972 | **0.038** |  |  |  |
| **Total bite rate excluding roving herbivores** | **Source of variation** | **df** | **MS** | **Pseudo-F** | ***p*-value** |
| **Main Test** | Site | 2 | 58.784 | 1.525 | 0.270 |
|  | Locality (Site) | 8 | 40.324 | 3.985 | **0.001** |


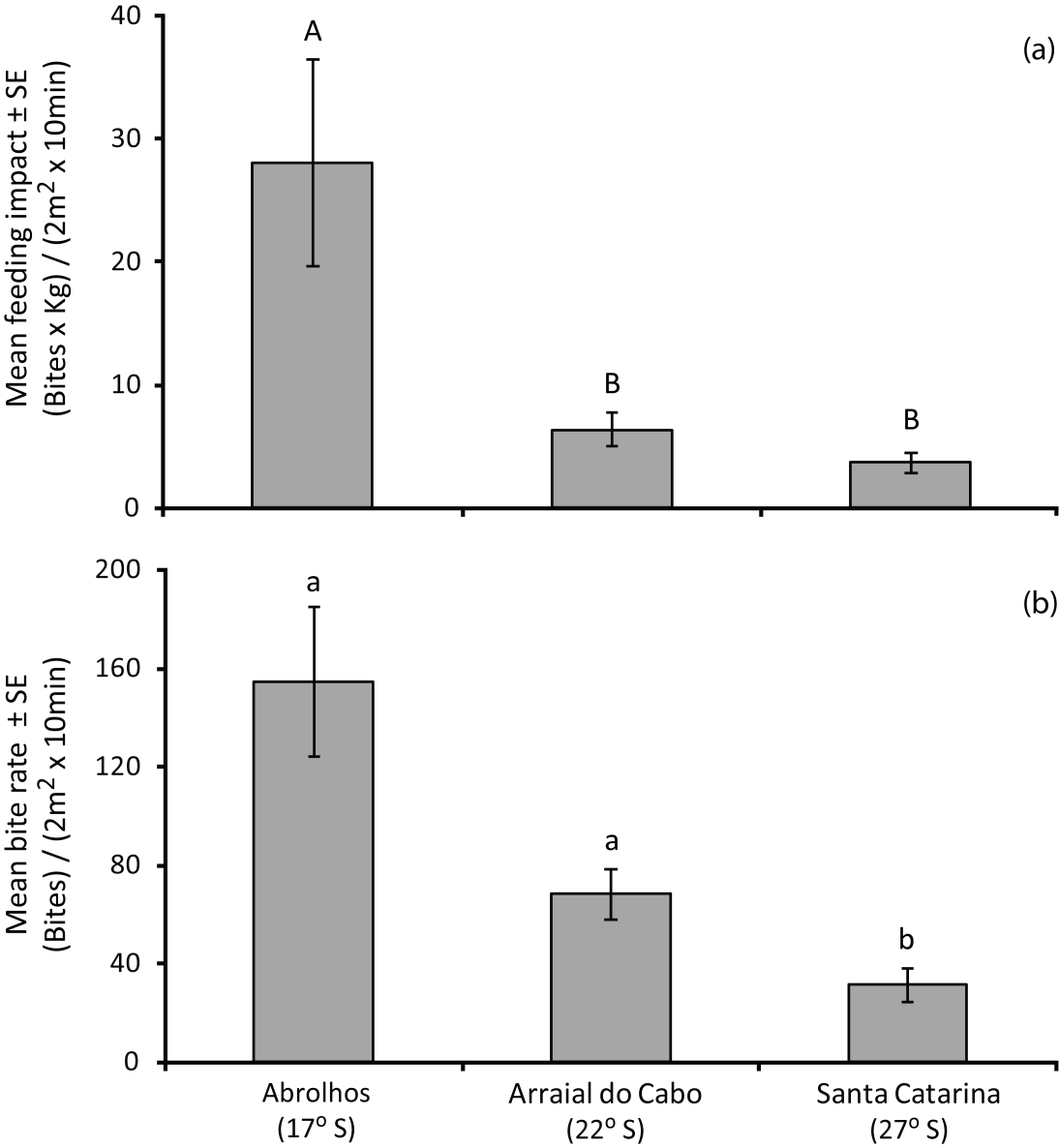


**Figure S1.** Mean feeding pressure (a) and non-mass-standardized bite rates (b) between the studied sites. Letters above the bars refers to pairwise comparisons from PERMANOVA tests, with upper case letters referring to the results in Table 1 and lower case letters to Table S3.

**
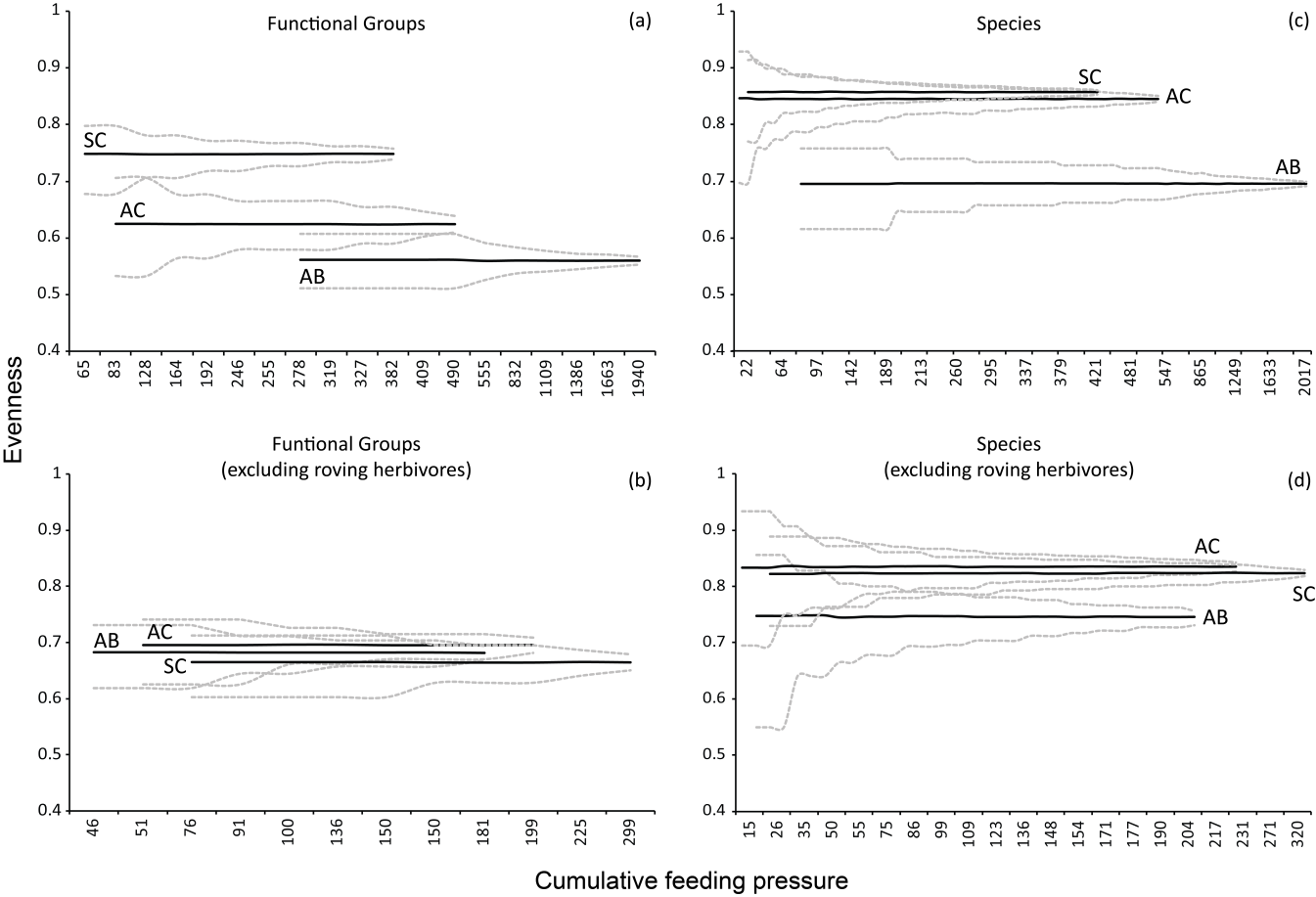
**

**Figure S2.** Cumulative rarefaction curves of the evenness of feeding pressure by functional groups (a, b) and species (c,d), accounting for all functional groups (a,c) and excluding roving herbivores (b,d). Gray dashed lines indicate 95% confidence intervals generated through 1000 iterations. AB = Abrolhos (17° S); AC = Arraial do Cabo (22° S); SC = Santa Catarina (27° S).
