# Supplementary figures and images for: Herbivory drives large-scale spatial variation in reef fish trophic interactions
Source: Ecol Evol. 2014 Nov 22;4(23):4553–66. doi: 10.1002/ece3.1310 (PMC4264904; doi:10.1002/ece3.1310)

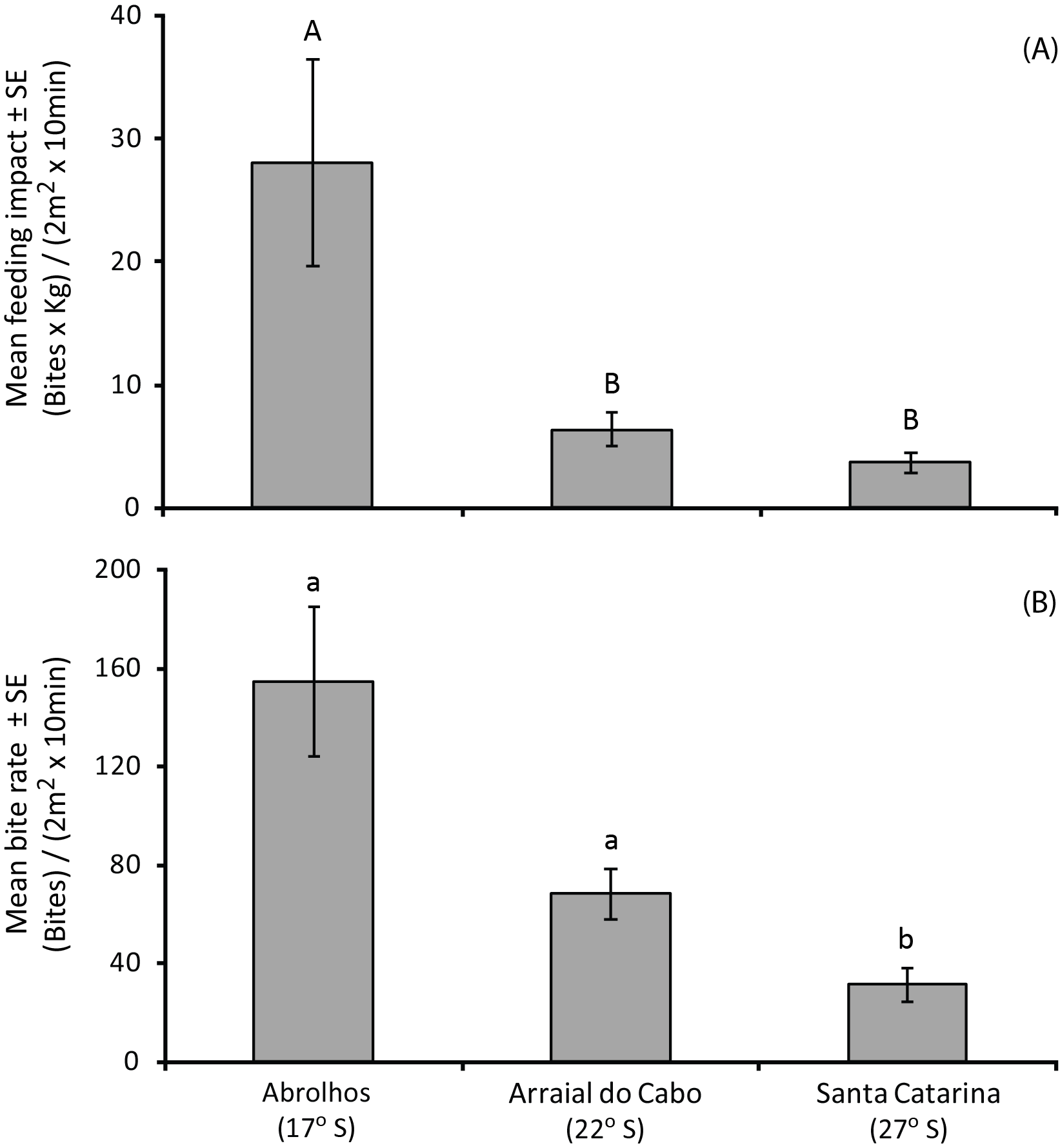

Supplement: Figure S1 — Mean feeding pressure and non-mass-standardized bite rates between the studied sites. [file ece30004-4553-sd3.tif]

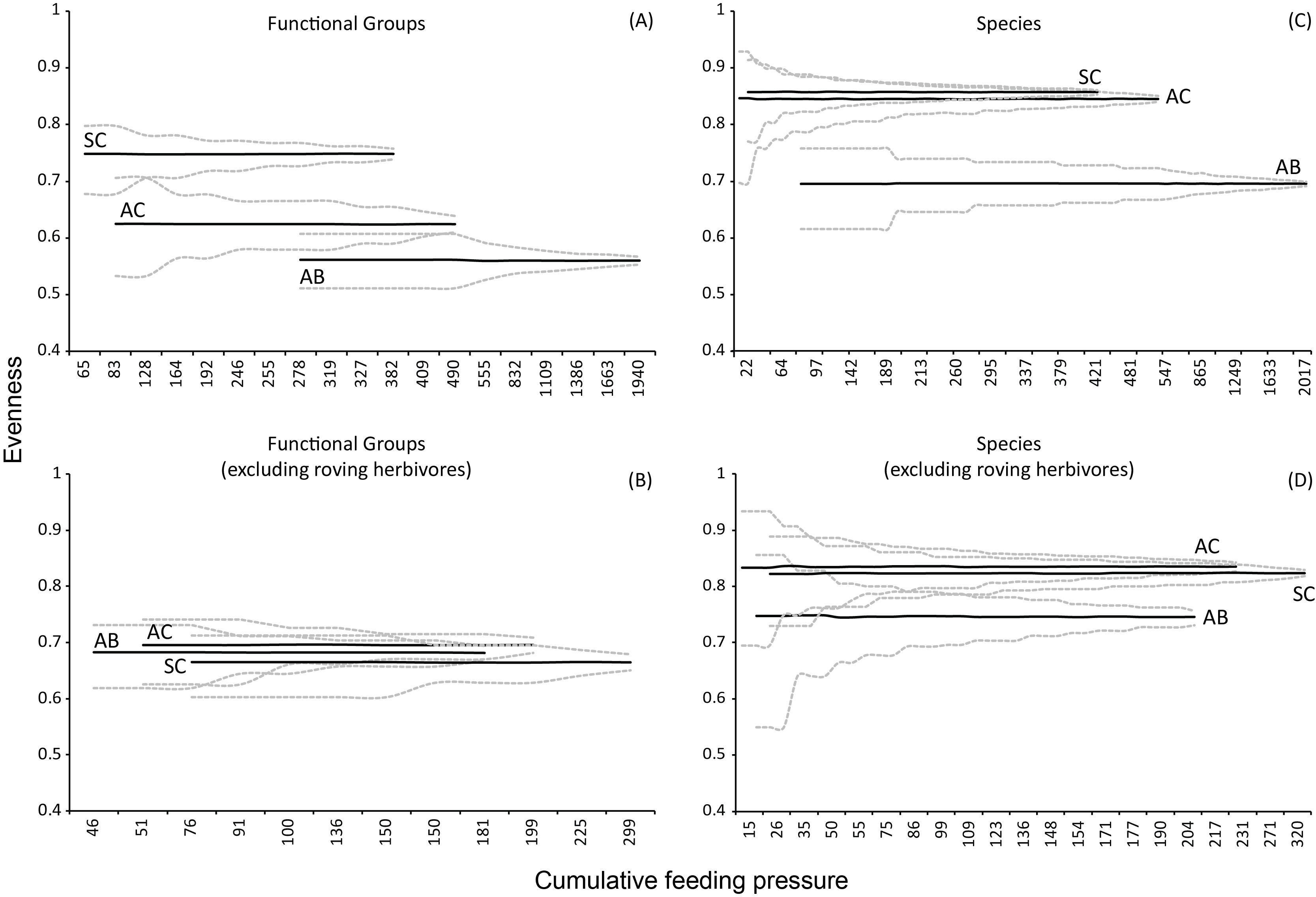

Supplement: Figure S2 — Cumulative rarefaction curves of the evenness of feeding pressure within functional groups and species, with and without roving herbivores, and comparisons between the studied sites. [file ece30004-4553-sd4.tif]
